# Distance and Sex Determine Host Plant Choice by Herbivorous Beetles

**DOI:** 10.1371/journal.pone.0055602

**Published:** 2013-02-06

**Authors:** Daniel J. Ballhorn, Stefanie Kautz, Martin Heil

**Affiliations:** Department of Botany/Plant Ecology, University of Duisburg-Essen, Essen, Germany; University of Bristol, United Kingdom

## Abstract

**Background:**

Plants respond to herbivore damage with the release of volatile organic compounds (VOCs). This indirect defense can cause ecological costs when herbivores themselves use VOCs as cues to localize suitable host plants. Can VOCs reliably indicate food plant quality to herbivores?

**Methodology:**

We determined the choice behavior of herbivorous beetles (Chrysomelidae: *Gynandrobrotica guerreroensis* and *Cerotoma ruficornis*) when facing lima bean plants (Fabaceae: *Phaseolus lunatus*) with different cyanogenic potential, which is an important constitutive direct defense. Expression of inducible indirect defenses was experimentally manipulated by jasmonic acid treatment at different concentrations. The long-distance responses of male and female beetles to the resulting induced plant volatiles were investigated in olfactometer and free-flight experiments and compared to the short-distance decisions of the same beetles in feeding trials.

**Conclusion:**

Female beetles of both species were repelled by VOCs released from all induced plants independent of the level of induction. In contrast, male beetles were repelled by strongly induced plants, showed no significant differences in choice behavior towards moderately induced plants, but responded positively to VOCs released from little induced plants. Thus, beetle sex and plant VOCs had a significant effect on host searching behavior. By contrast, feeding behavior of both sexes was strongly determined by the cyanogenic potential of leaves, although females again responded more sensitively than males. Apparently, VOCs mainly provide information to these beetles that are not directly related to food quality. Being induced by herbivory and involved in indirect plant defense, such VOCs might indicate the presence of competitors and predators to herbivores. We conclude that plant quality as a food source and finding a potentially enemy-free space is more important for female than for male insect herbivores, whereas the presence of a slightly damaged plant can help males to localize putative mating partners.

## Introduction

The factors that drive plant-herbivore interactions are complex and we are only beginning to understand their functional interplay in natural systems [1]. In addition to quantitative and qualitative variation in plant primary and secondary metabolites, the attractiveness of host plants to insect herbivores is influenced by multiple factors, such as the presence of phytopathogens, predators, parasitoids, soil biota, as well as of other conspecific and heterospecific herbivores [1]–[7]. Furthermore, plant attractiveness is affected by microclimatic conditions, plant architecture, and abundance [2]–[5]. The success of an herbivore in identifying a suitable host depends on its ability to locate the plant, assess leaf quality, utilize available nutrients, and cope with plant defensive traits [8], [9]. Not surprisingly, males and females are subject to different selective forces in this context. For females, an already damaged host plant releasing herbivore-induced volatile organic compounds (VOCs) might indicate a resource for which competition is high. According to the preference–performance hypothesis (PPH) [10], [11], females of insect species with herbivorous larvae select host plants with respect to their potential nutritive quality for their offspring [11]. In particular, the PPH relates to insects whose larvae have limited or no ability to search for alternative food plants, and are thus reliant on the mother's host plant choice [12]. In this line, females should also be more sensitive to variation of non-volatile ( = short-distance) plant cues such as primary and non-volatile secondary plant compounds, surface structures, tissue toughness, and water content; together these traits affect the overall nutritive value of the host plants. By contrast, males undergo higher pressure to localize mating partners. For male insect herbivores, the volatiles released from a damaged plant potentially indicate a suitable place for finding mates. Therefore, long- and short-distance signals might have very different effects on the host selection of males and females [9].

Sex-specific differences in the responses of herbivorous insects to plant VOCs have been reported for numerous insects belonging to various orders such as Lepidoptera [13]–[15], Coleoptera [16], [17], Diptera [18], and Heteroptera [19]. Similarly, gender dependent responses to short-distance cues are frequently reported, e.g., for Lepidoptera [20], and Coleoptera [21]–[23]. However, experimental evidence for sex-specific concerted effects of long- and short-distance cues in plant-herbivore systems is surprisingly scarce. As plants generally show a suite of traits affecting their attractiveness to herbivores, integrative studies are required to better understand the functional interplay of multiple factors, which drive the complex interactions in plant-herbivore systems.

To contribute in filling this gap in the understanding the sex-specific function of long- and short-distance plant cues, we used males and females of two chrysomelid herbivores (*Gynandrobrotica guerreroensis* and *Cerotoma ruficornis*). In their natural habitat in southern Mexico, these oligophagous beetles feed on a small range of legumes, but with distinct preference for wild lima bean (*Phaseolus lunatus*). We used choice experiments to analyze the response of the insects over long and short distances to cues of lima bean plants. We focused on two plant traits, i) the release of induced VOCs and ii) the constitutive cyanogenic potential (HCNp) of leaves. The HCNp means the total amount of toxic cyanide (CN^−^), which can be released from preformed cyanogenic compounds (in lima bean these compounds are the cyanogenic glycosides linamarin and lotaustralin) in response to cell damage. The induced VOCs have the potential to act over long distances. The HCNp, however, acts as a direct chemical defense that affects the feeding herbivore locally when free cyanide is released during the feeding process. In the present study, we experimentally induced the release of plant VOCs by application of jasmonic acid (JA) to lima bean foliage. Jasmonic acid is the central signaling molecule of the octadecanoid pathway [24], which is involved in regulation of induced plant defenses against herbivores [25], [26]. External application of aqueous JA-solution induces various defense mechanisms, including the release of VOCs [27].

In contrast to VOCs, the HCNp is not affected by JA application [28]. The ability to release cyanide from cyanogenic precursors in response to damage (cyanogenesis) is widespread in the plant kingdom. More than 4000 plant species are cyanogenic and this trait usually functions as direct defense against herbivores, but see [29], [30]. The release of herbivore-induced VOCs is even more common among plants. Many, if not all, plant species release VOCs in response to damage inflicted by herbivores [25], [31], [32]. These VOCs have a diverse array of ecological effects. VOCs can be used by arthropod parasitoids and predators in their search for hosts and prey insects, and thus function as an indirect defense mechanism for the plant [33]. Furthermore, released VOCs can induce or prime defenses in neighboring plants [34]–[36]. In lima bean for example, induced VOCs can enhance the secretion of carnivore-attracting extrafloral nectar in neighboring plants (plant-plant signaling) and other leaves of the same plant (within-plant signaling) [37]. Both of these indirect defenses, VOCs and EFN, significantly reduce herbivore damage under field conditions by attracting carnivorous arthropods [38]. In other studies, VOCs released from induced plants have also been shown to reduce feeding by insect herbivores (*Phyllotetra* spp.) [39], act as a repellent (*Gynandrobrotica guerreroensis*) [27], and reduce herbivore abundance (*Epitrix hirtipennis*) [40]. However, these VOCs represent openly presented information, and therefore can be used by the herbivores themselves to localize their host plants in the same manner as arthropods of the third trophic level [19], [41]–[43]. In this way the indirect defense by VOCs can cause significant ecological costs for the plant [44], [45]. The question remains open, however, whether VOCs can serve as reliable indicators of those traits that are of vital interest for herbivorous insects, such as quality of the plant as a food source and the presence of competitors, enemies, and putative mating partners.

In the present study, we analyzed the response of male and female chrysomelid beetles (*G. guerreroensis* and *C. ruficornis*) to VOCs released from wildtype lima bean plants at different stages of development (mature and young plants) and different levels of induction ranging from little to strongly induced. We determined the long-distance responses of males and females to VOCs both in an olfactometer and in a large cage. The same individual beetles were consecutively used in feeding trials to assess their short distance choice behavior. The HCNp of the same leaves was determined as an important direct defense of lima bean [28], [46], [47]. Our experimental setup thus allowed us to determine factors affecting different steps in the overall decision process in host selection among sex, VOCs and direct defense. To our knowledge, this experimental approach using the same individual beetles and plants in long- and short-distance choice experiments has never been conducted before.

Based on the aforementioned considerations, we predicted that the intensity of induction should determine beetle behavior. Furthermore, we hypothesized that factors directly determining food quality should be strong predictors of female behavior, whereas VOCs as a potential indicator of the presence of conspecific females should positively affect long-distance behavior of males. In contrast, females should negatively respond to VOCs as these may indicate competition and thus, reduced resources for the developing offspring.

## Methods

### Plants and Insects

Experiments were conducted from August to October 2006 in the coastal area of Oaxaca, Mexico. We conducted experiments with chrysomelid herbivores and wild lima bean plants. Plants and insects were collected directly before the experiments from a natural population 10 km W of Puerto Escondido (15°55′31.80″N, 97°9′4.68″W, 8 m a.s.l.). To consider potential effects on herbivores arising from ontogenetic variation of plant traits, experiments were carried out with shoot parts of mature plants as well as with young, intact potted plants that both were derived from the same site. Potted plants were grown from seedlings which were collected when unfolding their primary leaves. Seedlings were cultivated (3–4 weeks) in 250 ml plastic pots filled with soil from natural sites. Plants were exposed to natural conditions, watered twice a day and fertilized once a week with nitrogen-phosphate fertilizer (Blaukorn®-Nitrophoska-Perfekt, Compo GmbH & Co. KG, Münster, Germany) in a concentration of 0.25 mg L^−1^ (80 mL solution per plant). Herbivorous insects naturally approaching the plants were manually removed twice a day to avoid any uncontrolled induction of herbivore-induced volatiles. At the time of the experiments, plants were 60–90 cm in height and had developed 10 to 15 leaves.

We used the phyllophagous beetles *Cerotoma ruficornis* Olivier and *Gynandrobrotica guerreroensis* Jacoby (Chrysomelidae: Galerucinae: Luperini: Subtribe Diabroticina) as herbivores. Beetles were randomly collected from host plants to represent natural ratios of sexes and ages. The insects were kept in transparent 250 ml plastic cups and were deprived of food for 1 d prior the experiment (water supplied on cotton). The sex of individual beetles was identified (based on sexual organs and presence/absence of ovaries) after the experiments using a binocular microscope. In addition to locusts (Acrididae, Pyrgomorphidae) and larvae of the bean leaf roller (Hesperidae: *Urbanus proteus* L.), these chrysomelid species were the most abundant herbivores on lima bean at the study site in the period of August to October. They were present throughout the day, exhibiting two peaks of feeding and moving activity in the first hours after dawn (8:00 a.m.–10:00 a.m.) and dusk (8:00 p.m.–11:00 p.m.) (pers. observ.). Chrysomelid species were identified by Astrid Eben (Instituto de Ecología, Veracruz, Mexico).

### Experimental Treatment of Plants

Herbivore-induced volatile organic compounds (VOCs) were induced by spraying foliage with aqueous solutions of jasmonic acid (JA) between 08:00 and 09:00 a.m. Plants used for VOC collection and choice experiments were treated identically and were randomly selected for the respective experiment. Control plants were treated the same way, but were sprayed with water instead of JA solution. We used different JA concentrations (aqueous solutions of 0.001, 0.01, 0.1 and 1.0 mmol L^−1^ JA) to analyze quantitative effects of experimental induction on VOC emission. Leaves were sprayed with JA solutions until completely moistened and allowed to dry (10 min) before being sprayed a second time. Plants were allowed to dry again and placed into PET bags (“Bratenschlauch”, Toppits, Minden, Germany, a PET foil that does not emit detectable amounts of volatiles even under exposure to temperatures of up to 150°C) for volatile collection [28]. Bagged plants were watered and received a natural photoperiod without being exposed to direct sunlight. Temperature in the bags was controlled and did not exceed 32°C.

Experimental treatment of mature shoot parts was the same as for potted plants. However, shoots were sprayed in the field before being cut off from the plants. Shoots were placed in bags of PET foil (“Bratenschlauch”) and immediately transported to the field laboratory for further experiments. Plant material was transported under cooled conditions (<20°C) and water supply. Cutting off the shoots did not induce any detectable release of volatiles (Ballhorn, unpubl. data).

In addition to quantitative effects of JA-induced VOC emission on herbivore behavior, we analyzed the effects of VOCs released following insect leaf damage. For these experiments we used 10 adult beetles (*Gynandrobrotica guerreroensis*). Beetles were placed on potted plants and shoots between 08:00 and 09:00 a.m. Plant material was bagged with nets and beetles were allowed to feed on foliage for 24 h. The following day between 08:00 and 09:00 a.m., beetles and nets were removed and plants were bagged in PET foil for VOC collection. Experimental setup and number of treated and control plants (resp. shoot parts) were the same as described for experiments with JA.

### Olfactometer Experiments

Beetle behavior in response to VOCs released from JA-treated or herbivore-damaged plants was tested in a Y-olfactometer. The glass olfactometer consisted of a stem (length 16 cm) and two arms (length 13 cm, inside angle 60°). Glass tubes had an inner diameter of 4 cm. Inflowing air was cleaned by charcoal filters (1.5 mg of charcoal, CLSA-Filters, Le Ruissaeu de Montbrun, France) and then passed over the plants placed in PET foil bags for 1 h prior the experiment. Constant air-flow (ca. 750 mL min^−1^) was provided by a ventilator at the end of the olfactometer. All experiments were conducted between 06:00 p.m. and 12:00 a.m. at a temperature of 28–30°C (air humidity >90%). Light conditions were standardized by placing the entire setup in a dark room and directional movement of the beetles was ensured by centering one lamp (40 W) 50 cm in front of the Y-olfactometer. In the olfactometer, individual beetle choice was recorded. Each beetle was tested eight times, in order to calculate a percent decision that allowed a direct and quantitative comparison with the data obtained in feedings trials. Only beetles entering one of the arms within 3 min were counted as having made a decision. We used different concentrations of jasmonic acid for induction of VOCs (1.0, 0.1, 0.01, and 0.001 mmol L^−1^). The following choice situations were created for both mature shoot parts and potted plants: I (1.0):C, I (0.1):C, I (0.01):C and I (0.001):C. In addition to JA-induced lima beans, we used plants that were induced by herbivore-damage (HI; herbivore-induced) and tested these plants against controls (HI∶C). We conducted controls, in which every beetle had the choice between an empty arm and a control shoot or plant (0∶C) as well as two empty arms (0∶0). In further controls, we tested induced shoots and induced intact young plants against each other [for each type of plant material: I (1.0)∶I (1.0)] as well as untreated shoots and plants (for each type of plant material: C∶C), in order to evaluate potentially different effects of plant material (i.e., potted young plant or mature shoots) on choice behavior of insects. To prevent any bias due to possible unforeseen asymmetries in the experimental setup, after every ten trials, the odor source was changed and the odorant and control arms were reversed. Before changing the odor source, the glass tubes were washed with water and detergent, and then wiped with hexane and acetone. Olfactometer experiments were conducted over seven consecutive weeks between August and October 2006, each with different sets of plants and different sets of beetles. Altogether, *n* = 406 individuals of *C. ruficornis* (males: *n* = 187, females: *n* = 219) and *n* = 389 individuals of *G. guerreroensis* (males: *n* = 188; females: *n* = 201) were used in these experiments.

### Feeding Experiments

Feeding experiments were conducted using the same beetle individuals and plant material as in the previously conducted olfactometer experiments. Single beetles were placed in 250 ml plastic cups, each containing one leaflet of each type of plant material from the olfactometer experiments creating a binary choice situation. We used completely intact leaflets of similar developmental stages for the feeding trials to reduce potential effects of ontogenetic variability in leaf structure. After 24 h, leaflets were digitally photographed (Camedia C-4000 Zoom, Olympus, Hamburg, Germany) on a scale to calculate missing leaf area using the analySIS® software (Olympus Soft Imaging Solutions GmbH, Münster, Germany). After the feeding experiments, remaining leaf material was subjected to HCNp analysis (see below). In the feeding experiments we used *n* = 314 individuals of *C. ruficornis* (males: *n* = 146, females: *n* = 168) and *n* = 299 individuals of *G. guerreroensis* (males: *n* = 148; females: *n* = 151). The number of beetles in olfactometer experiments does not match the number of beetles in feeding trials because additional beetles were used in the olfactometer for control experiments such as empty arms (0∶0) and empty arms vs. control plants (0∶C).

### Free-Flight Experiments

Free-flight choice experiments were conducted in a cage (length 1.25× width 0.70× heights 1.00 m) between 3:00 and 6:00 p.m. in the open. We used young potted plants, which were placed at each end of the cage with a distance of 1 m. Beetles were placed in the middle between both plants through a small opening in the front. Each beetle was tested individually eight times, and only beetles landing on one of the plants within 10 min were considered as having made a decision. Control versus JA-induced plants were tested at two different concentrations [I (1.0)∶C and I (0.001)∶C] as well as control vs. herbivore-damaged plants (HI∶C). In a control setup two undamaged plants were tested against each other (C∶C). In free-flight experiments we used *n* = 66 individuals of *C. ruficornis* (males: *n* = 33, females: *n* = 33) and *n* = 71 individuals of *G. guerreroensis* (males: *n* = 37; females: *n* = 34). When choice experiments were finished, young leaves of the experimental plants were used for feeding experiments with individual beetles and HCNp as well as leaf area consumption were determined as described above.

### Gas Chromatography-Mass Spectrometry

Plant volatiles (VOCs) that were released from mature shoots and young potted plants in response to the different treatments were collected in a closed-loop stripping system (24 h) on charcoal filters (1.5 mg of charcoal, CLSA-Filters, Le Ruissaeu de Montbrun, France). Volatiles were dissolved from the charcoal filters with dichloromethane to which n-bromodecane (200 ng µL^−1^) had been added as an internal standard (IS) [48]. Samples were transferred in glass capillaries (disposable micropipettes with ring mark; Blaubrand® intraMARK, Buddeberg GmbH, Mannheim, Germany), and then subjected to GC-Trace-MS (Trace GC Ultra DSQ; Thermo Electron, Austin, TX). The program for separation [Rtx5-Ms column (Restek, Philadelphia, PA), 15 m×0.25 mm; 0.25 µm coating] was 40°C initial temperature (2 min), 10°C min^−1^ to 200°C, then 30°C min^−1^ to 280°C with He (constant flow 1.5 mL min^−1^) as carrier gas.

### Cyanogenic Features of Plant Material

**Figure 1:**
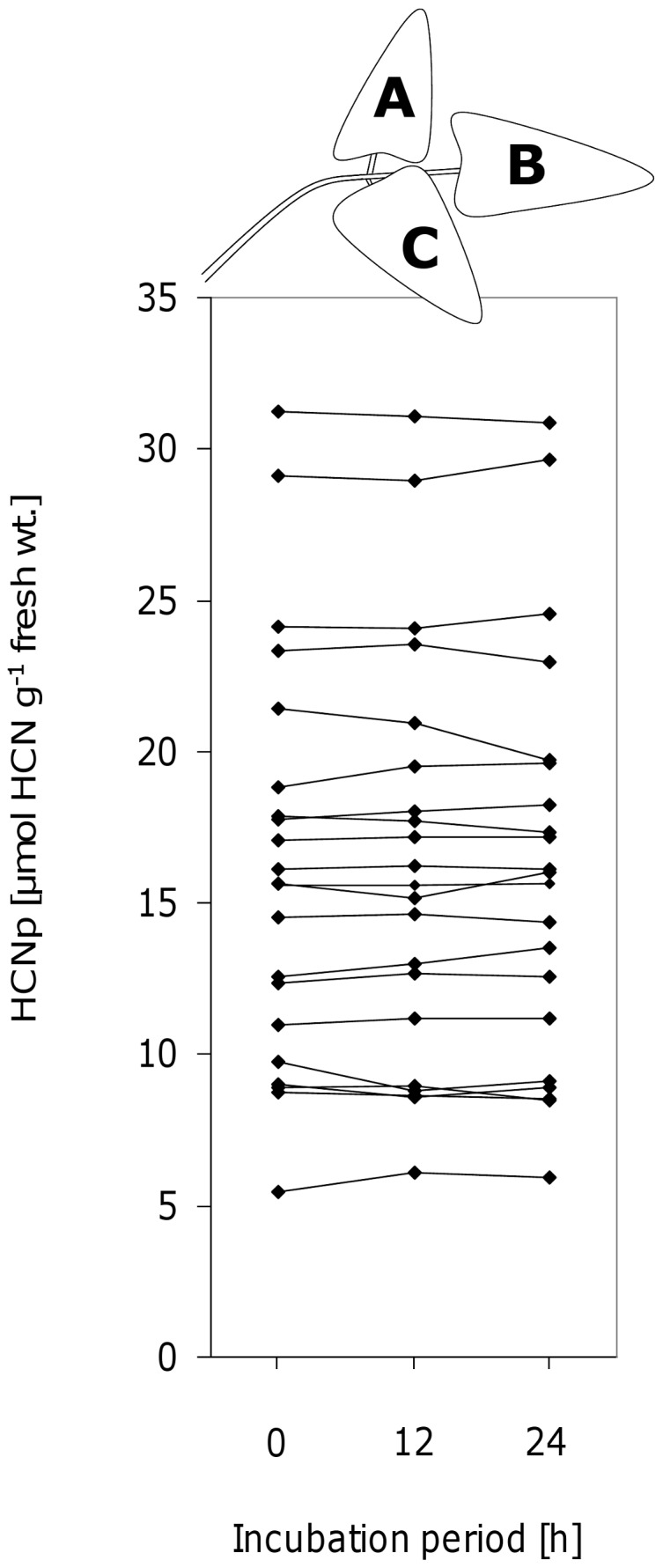
Effect of experimental period on cyanogenic potential (HCNp). Three leaflets (A,B,C) of individual lima bean leaves (*n* = 21 leaves each from different plants) were analyzed for HCNp after 0 (fresh), 12, and 24 h. Within individual trifoliate leaves no significant differences of HCNp could be observed for the leaflets after different experimental periods according to Friedman-Test (χ^2^ = 1.71, d.f. = 2, *P* = 0.918).

The cyanogenic potential (HCNp; maximum amount of cyanide that can be released from a given tissue) of leaf material was quantified by enzymatic degradation of cyanide containing precursors (i.e., cyanogenic glycosides linamarin and lotaustralin) by adding specific β-glucosidase from rubber tree, *Hevea brasiliensis* [22]. The released hydrogen cyanide was spectrophotometrically quantified (585 nm) using the Spectroquant® cyanide test (Merck, Darmstadt, Germany). The method is described in detail in Ballhorn et al. [49]. Since HCNp was analyzed after leaflets had been exposed to herbivore damage for an experimental period of 24 h, we tested whether or not HCNp of leaflets decreased under experimental conditions over this time period. In this experiment, the three leaflets of individual trifoliate leaves each were analyzed for HCNp after 0, 12, and 24 h. Within individual trifoliate leaves (*n* = 21), the position of leaflets analyzed after the different time periods was set at random (Fig. 1).

### Statistics

The differences among different jasmonic acid (JA) treatments in the amounts of released VOCs were analyzed with a *post hoc* analysis (Tukey's HSD; *P*<0.05) after univariate ANOVA. To test for significant differences of VOC emissions of mature shoot parts and young intact plants within one treatment group, Wilcoxon signed-rank tests were applied. Friedman-tests were applied to test for time-depended changes in the cyanide concentrations in leaflets of individual trifoliate leaves after incubating the leaflets for 0, 12, and 24 h. In the Y-tube olfactometer experiments, a percentage decision of individual beetles for the different odor sources was calculated (each beetle had been subjected to the same experimental setup eight times). In the olfactometer and free-flight cage experiments, the differences in the percentage decisions of individual beetles between induced (1.0; 0.1; 0.01; 0.001 mmol L^−1^ JA; herbivore induction) plant material (mature shoots and potted young plants) and the controls were analyzed with Wilcoxon signed rank tests after arcsine transformation of data [50]. Correspondingly, in control experiments using untreated or identically treated plants (in olfactometer and cage experiments) or two empty arms (in olfactometer experiments), the percentage decision of beetles for both odor sources (or arms of the olfactometer) was calculated. Data on choice behavior of insects obtained from the control experiments were analyzed with Wilcoxon tests. Differences in choice behavior of beetles depending on sex were analyzed with Mann Whitney U-tests comparing male and female beetles for each experiment separately. To test for significant effects of the factors ‘Beetle species’ (i.e. *Cerotoma ruficornis* or *Gynandrobrotica guerreroensis*), ‘Sex of beetle’, and ‘Beetle species×Sex of beetle’ we used a general linear model (GLM). Correlations of insect choice behavior in olfactometer and free-flight experiments with actual feeding decisions in subsequent feeding trials were analyzed with Pearson's correlations. For statistical analyses we used SPSS (IBM SPSS Statistics 19).

## Results

### Volatile Organic Compounds (VOCs)

**Figure 2:**
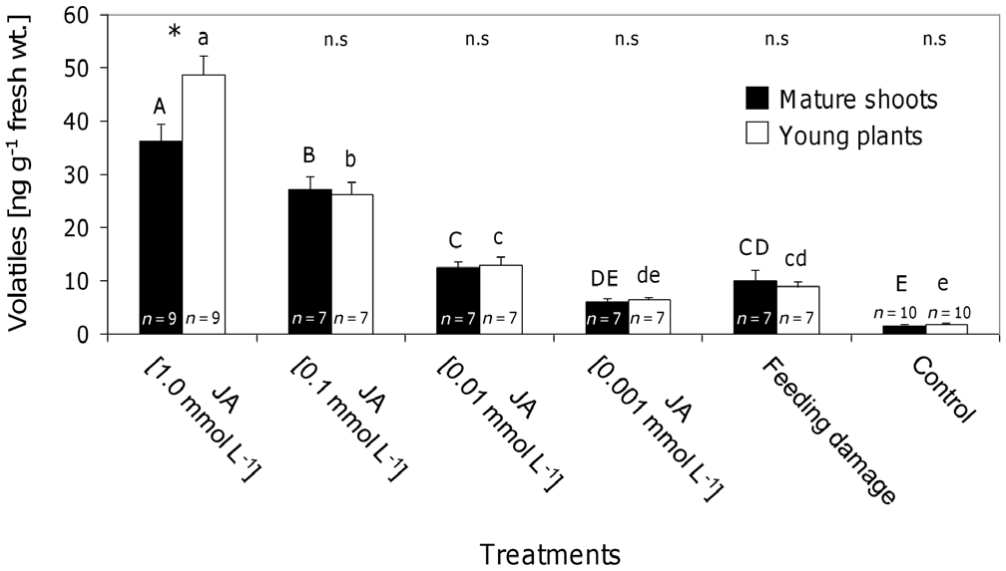
Effect of dose of jasmonic acid, herbivore damage and plant developmental stage on the release of volatiles. Mature shoots and young entire plants were treated with different jasmonic acid (JA) concentrations (1.0, 0.1, 0.01, and 0.001 mmol L^−1^) or were damaged by herbivore (*Gynandrobrotica guerreroensis*) feeding. Among treatments, significant differences [according to *post hoc* analysis (LSD, *P*<0.05) after one-way ANOVA] in VOC release are indicated with different capital (shoots) or small-typed (young plants) letters at top of the columns. ‘*’ (*P* = 0.029) and ‘n.s’ at the upper panel of the figure indicate significant resp. non-significant difference in VOC release between shoots and young plants (according to Mann-Whitney-U Test). Values represent means ± SD. *n* of repetitions is indicated in the respective columns.

Both mature shoot parts and young intact lima bean plants showed induced release of VOCs in response to jasmonic acid (JA) treatment (Fig. 2). The amounts of released volatiles depended on concentrations of JA applied. Jasmonic acid treatments of 1.0 mmol L^−1^ resulted in the highest release of VOCs for both mature shoots and potted plants, while induction with JA solutions at lower concentrations (0.1, 0.01 and 0.001 mmol L^−1^) resulted in lower amounts of VOCs released (Fig. 2). Plant material induced with the lowest concentration of JA released higher amounts of volatiles than the controls, however, differences were not significant (Fig. 2). For each type of plant material (i.e., mature shoots or young potted plants) differences in quantitative release of VOCs were significant among the different treatments [according to *post hoc* analysis (Tukey's HSD, *P*<0.05) after one-way ANOVA (mature shoots: *F*_5, 41_ = 55.530, *P*<0.001; young potted plants: *F*_5, 46_ = 98.246, *P*<0.001; Fig. 2]. Among the different treatments, the quantitative release of VOCs was not significantly different depending on type of plant material with exception of shoots and plants treated with 1 mmol L^−1^ JA (according to Mann-Whitney U-test; Fig. 2 and Table S1). Here, young potted plants showed a higher release of VOCs than mature shoots (Fig. 2). Amounts of volatiles released from the different types of plant material in response to herbivore damage ranged between the amounts of volatiles emitted following JA treatment at concentrations of 0.001–0.01 mmol L^−1^ (Fig. 2).

### Cyanogenic Potential (HCNp) of Leaves

Leaflets of mature shoot parts and young intact plants each showed substantial quantitative variability of HCNp. Concentration of cyanide-containing compounds in leaflets ranged from 0.93 to 79.98 (22.72±10.91; mean ± SD; *n* = 696) µmol HCN g^−1^ fresh wt. for shoots and 0.20 to 64.69 (23.04±11.19; mean ± SD; *n* = 740) µmol HCN g^−1^ fresh wt. for potted plants. Comparative analyses of leaflets of individual trifoliate leaves after 0, 12, and 24 h (exposed to natural photoperiod and temperature) revealed no significant differences of HCNp among leaflets depending on experimental time period (according to Friedman-Test, *n* = 21 leaves; Fig. 1).

### Olfactometer Experiments

**Figure 3:**
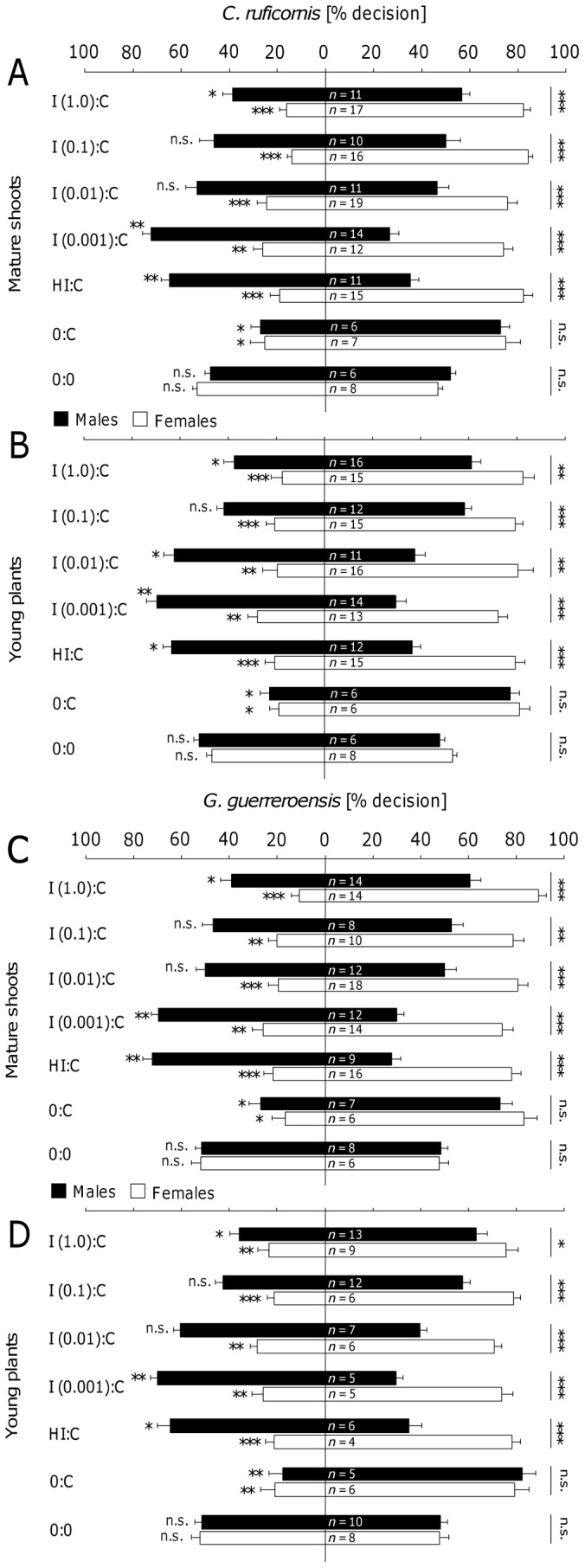
Choice behavior of beetles in the Y-tube olfactometer. In a series of olfactometer experiments with *Cerotoma ruficornis* (A,B) and *Gynandrobrotica guerreroensis* (C,D), shoots of mature lima bean plants (A,C) and young entire plants (B,D) were used. The seven setups tested were: JA-induced plants (I; the four tested JA concentrations were 1.0, 0.1, 0.01, and 0.001 mmol L^−1^) and herbivore-induced (HI, induced by *G. guerreroensis*) vs. control plants (sprayed with water instead of JA). As controls, beetles were given the choice to select between an empty olfactometer arm and control plants (0∶C) as well as between two empty olfactometer arms (0∶0). Bars represent means ± SE of percentage decisions made by each sex of beetles. The number of beetles tested in the different setups is indicated for each gender. Every beetle was subjected to the same experimental setup 8 times and the proportions of decisions were used for further analyses. Asterisks at the left side of the columns represents significant differences in percentage decisions between both odor sources according to Mann-Whitney U-test (* = *P*<0.05; ** = *P*<0.01; *** = *P*<0.001). Asterisks at the right side of the columns represent significant differences in choice behavior between male and female beetles according to Wilcoxon signed-rank test (* = *P*<0.05; ** = *P*<0.01; *** = *P*<0.001).

In olfactometer experiments, *C. ruficornis* and *G. guerreroensis* (Fig. 3) of both sexes significantly preferred controls compared to plant material induced with JA at high (1.0 mmol L^−1^) concentrations (according to Wilcoxon test; Fig. 3 and Table S2). However, females showed significantly higher preference for the controls than males [according to Mann Whitney U-test]. These sex-depending differences in choice behavior became even more evident when beetles were exposed to volatiles released from plant material treated with lower JA concentrations (Fig. 3 and Table S2). Female beetles of *C. ruficornis* and *G. guerreroensis* still were significantly repelled by VOCs released from mature shoots (Fig. 3A,C) and intact young plants (Fig. 3B,D) treated with JA solutions of 0.1, 0.01 and 0.001 mmol L^−1^ as well as by VOCs released from plant material damaged by herbivores (H∶C). In contrast to females, males of both species showed no significant preference for both odor sources when they had the choice between plant material treated with 0.1 and 0.01 mmol L^−1^ JA and the respective controls. Males of *C. ruficornis*, however, represent an exception as they significantly preferred young intact plants induced with 0.01 mmol L^−1^ JA over the controls (according to Wilcoxon test; Fig. 3B). Given the choice to select between plant material treated with 0.001 mmol L^−1^ JA and plants damaged by herbivores, males of *C. ruficornis* and *G. guerreroensis* significantly preferred induced shoots and intact plants over the controls and therefore showed the opposite choice behavior compared to females. Thus, for males we observed a complete shift from rejecting highly induced plants (treated with 1 mmol L^−1^ JA) to attraction by plants induced with low concentrations of JA (0.001 mmol L^−1^ JA) and herbivore-damaged plants, whereas females consistently preferred the controls over induced plant material. However, among all experiments in which beetles of both species had the choice to select between JA treated and herbivore damaged plant material we observed significant sex-dependent differences even when both sexes showed the same qualitative response (according to Mann Whitney U-test).

**Figure 4:**
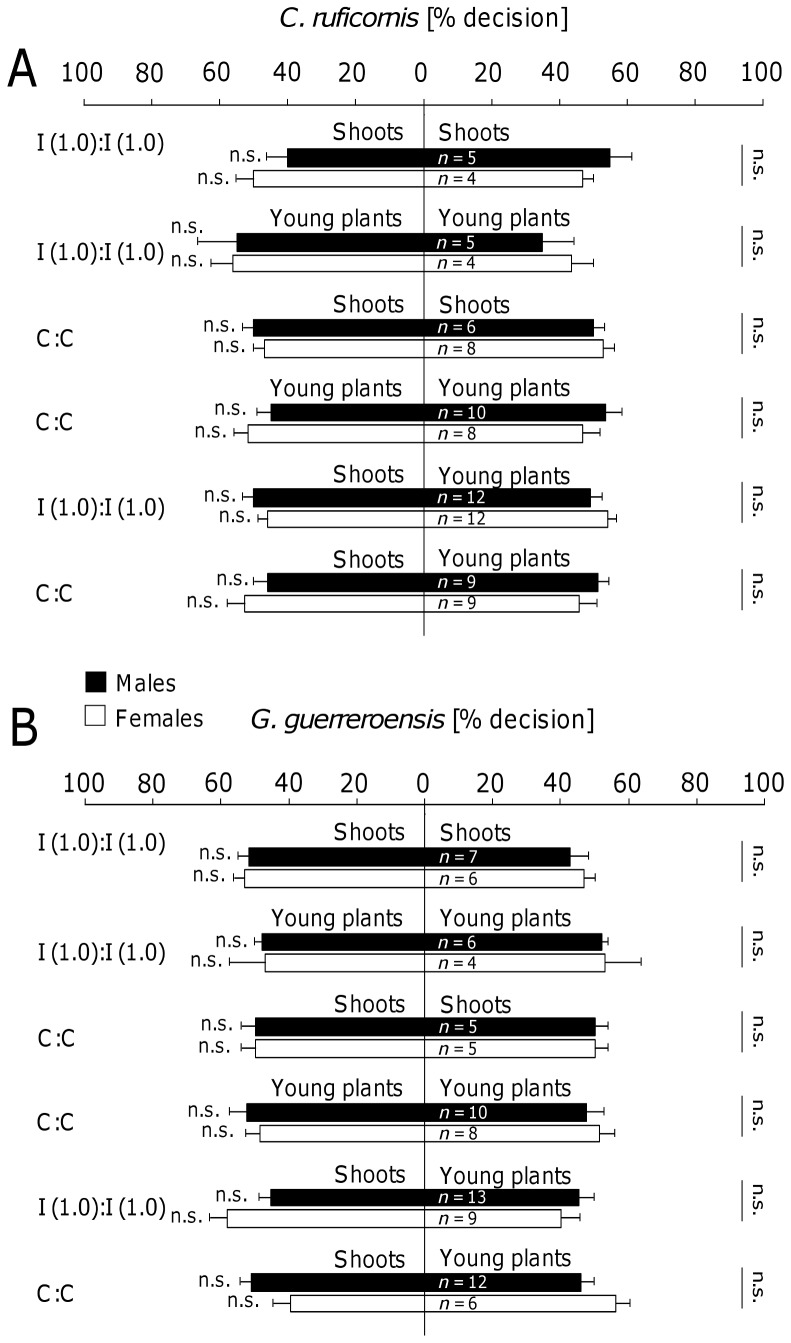
Control experiments in the Y-tube olfactometer. In a series of olfactometer experiments using *Cerotoma ruficornis* (A) and *Gynandrobrotica guerreroensis* (B), induced shoots and young plants as well as control shoots and plants (sprayed with water instead of JA) were offered to the herbivores. In addition, pairs of treated and untreated shoots and plants were offered to beetles. Bars represent means ± SE of percentage decisions made by each sex of beetles. The number of beetles tested in the different setups is indicated for each sex. Each beetle was subjected to the same experimental setup 8 times and portions of decision for each plant treatment were used for further analyses.

When given the choice to select between untreated plant material (from mature shoots or potted young plants) and an empty arm, beetles of both species and sexes significantly preferred plant material (according to Wilcoxon test; Fig. 3A–D). In a series of control experiments, we found no significant differences in beetles' decisions when the beetles had the choice to select between two empty arms [(0∶0); Fig. 3], two arms containing induced shoots or potted plants [I (1.0)∶ I (1.0)] or untreated plant material [(C∶C); Fig. 4 and Table S3].

### Free-Flight Experiments

**Figure 5:**
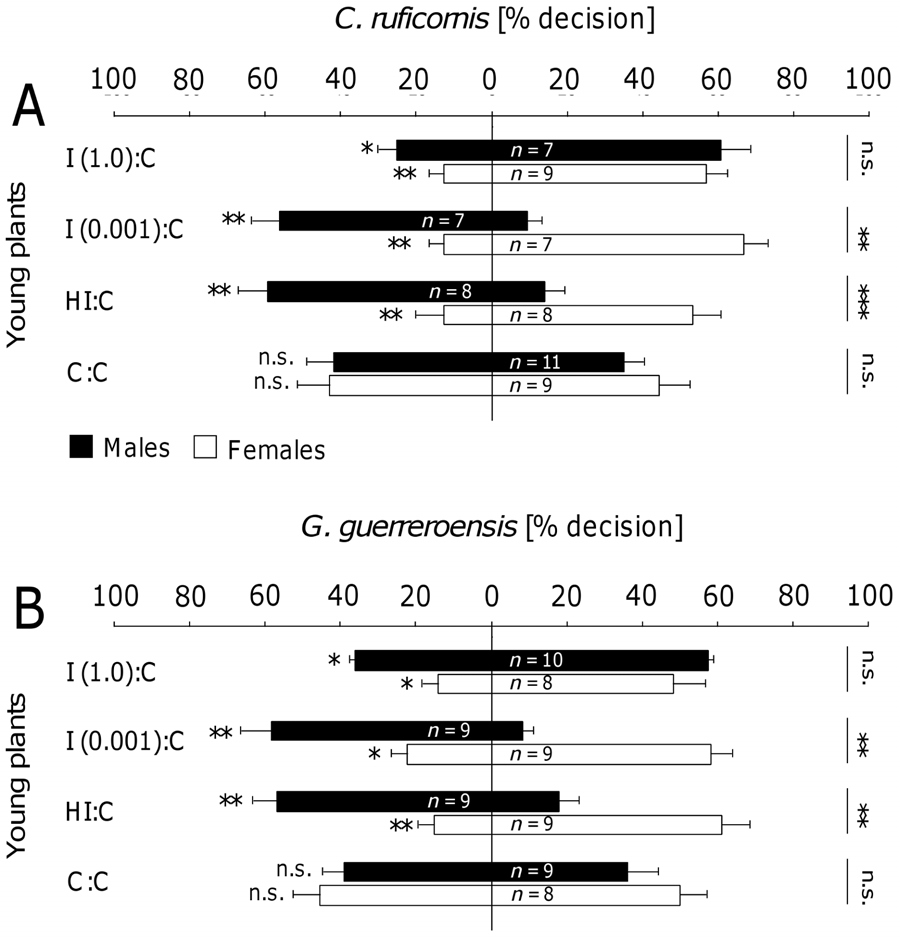
Choice behavior of beetles in free-flight experiments. In free-flight experiments with *Cerotoma ruficornis* (A) and *Gynandrobrotica guerreroensis* (B), young potted lima bean plants were offered and choice behavior of the insects was evaluated. Five different setups were included in the experiments, that is jasmonic acid (JA)-induced plants (I; JA concentrations: 1.0, 0.1, 0.01, and 0.001 mmol L^−1^) and herbivore-induced (HI) vs. control (untreated) plants. In both HI-experiments herbivore damage was invoked be *G. guerreroensis*. In addition, beetles were given the choice to select between control plants (C∶C). Each beetle was subjected to the same experimental setup 8 times and portions of decision for each plant treatment were used for further analyses. The number of beetles tested in the different setups is indicated for each sex. Asterisks at the left side of the columns represents significant differences in percentage decisions between both odor sources according to Mann-Whitney U-test (* = *P*<0.05; ** = *P*<0.01; *** = *P*<0.001). Asterisks at the right side of the columns represent significant differences in choice behavior between male and female beetles according to Wilcoxon signed-rank test (* = *P*<0.05; ** = *P*<0.01; *** = *P*<0.001).

Choice behavior of *Cerotoma ruficornis* and *Gynandrobrotica guerreroensis* in free-flight cages with pairs of induced and control plants was similar to choices made in olfactometer experiments. Beetles of both species and sexes significantly preferred controls over plants treated with high concentrations of jasmonic acid (1.0 mmol L^−1^). However, in contrast to olfactometer experiments, we found no significant differences in choice behavior between males and females when offering highly induced (1.0 mmol L^−1^) and control plants (according to Mann Whitney U-test; Fig. 5 and Table S4). When given the choice to select between controls and plants treated with low concentrations of jasmonic acid (0.001 mmol L^−1^ JA) and between herbivore-damaged and control plants (HI∶C), male beetles preferred induced plants over the controls, whereas females preferred the controls (Fig. 5). When offering pairs of untreated plants, beetles' decisions were evenly distributed (Fig. 5 and Table S5).

### Feeding Trials

**Figure 6:**
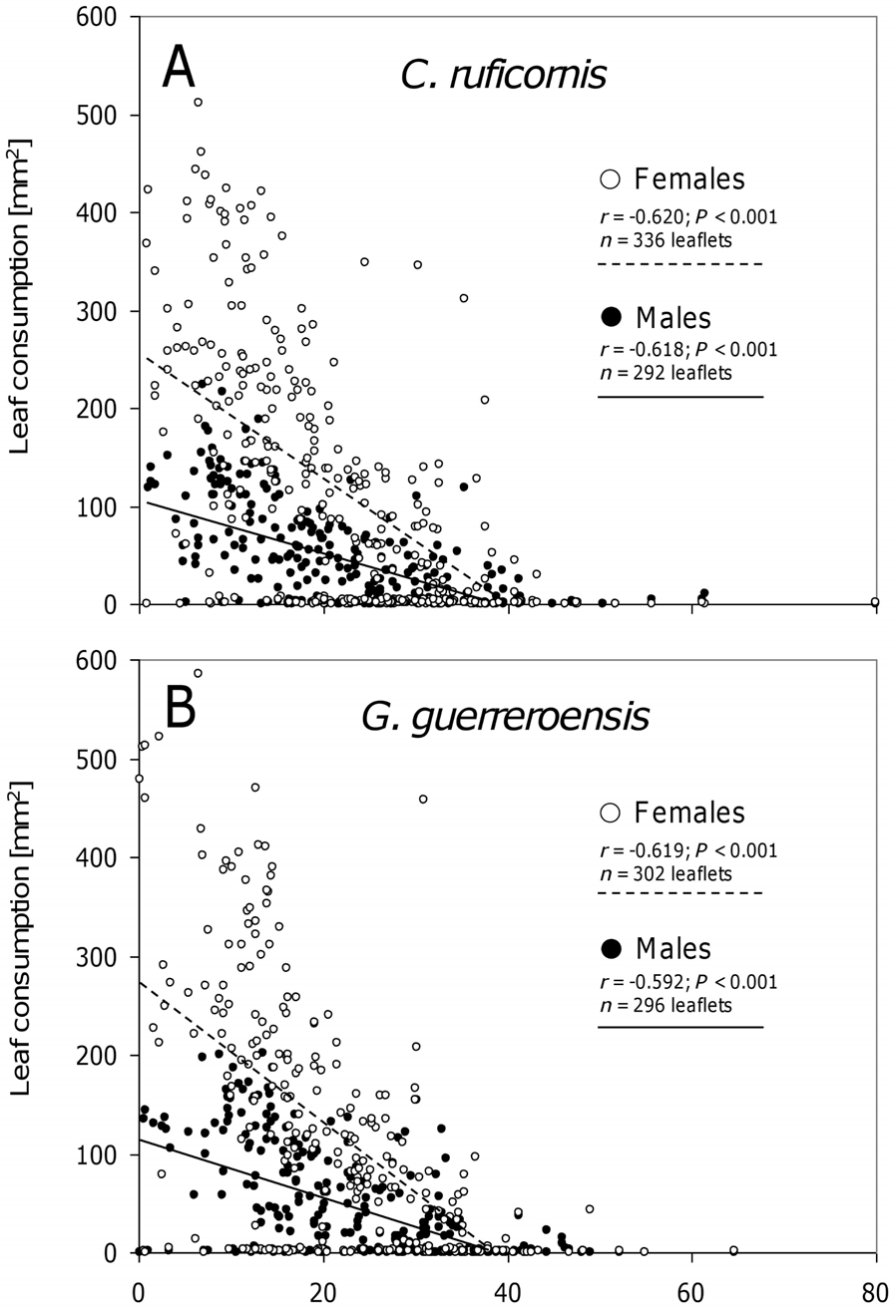
Leaf consumption of beetles in feeding trials. In binary-choice feeding experiments each two leaflets derived from plants previously used in olfactometer experiments were offered to individual beetles of *Cerotoma ruficornis* (A) and *Gynandrobrotica guerreroensis* (B) and leaf area consumption was determined. In this figure, it is not differentiated between leaf material derived from mature shoots or young entire plants. Pearson's correlations were calculated to evaluate effects of HCNp on leaf consumption.

Among all feeding trials, females of both species consumed substantially more leaf material than males (Fig. 6). Although overall leaf consumption of *Cerotoma ruficornis* males tended to be lower than for *Gynandrobrotica guerreroensis* males, females of both species consumed relatively similar amounts of leaf material to one another (Fig. 6). Consequently, general linear model (GLM) predicted a significant effect of ‘Sex of beetles’ but not of ‘Beetle species’ on consumed leaf area (Table 1).

**Table 1:** Effect of beetle species and sex of beetles on leaf area consumption.

Source	*SS*	d.f.	*F*	*P*-value
Beetle species	832.149	1	2.427	0.363
Error	342.881	1		
Sex of beetle	1312564.819	1	3828.044	<0.05
Error	342.881	1		
Beetle species×Sex of beetle	342.881	1	0.034	0.853
Error	1.217*10^7^	1221		

In feeding trials with *Cerotoma ruficornis* and *Gynandrobrotica guerreroensis*, we measured consumed leaf area by the insects. Results were analyzed using a general linear model (GLM) for analysis of variance with leaf area consumption as variable. ‘Beetle species’ and ‘Sex of beetle’ were set as fixed factors.

**Figure 7:**
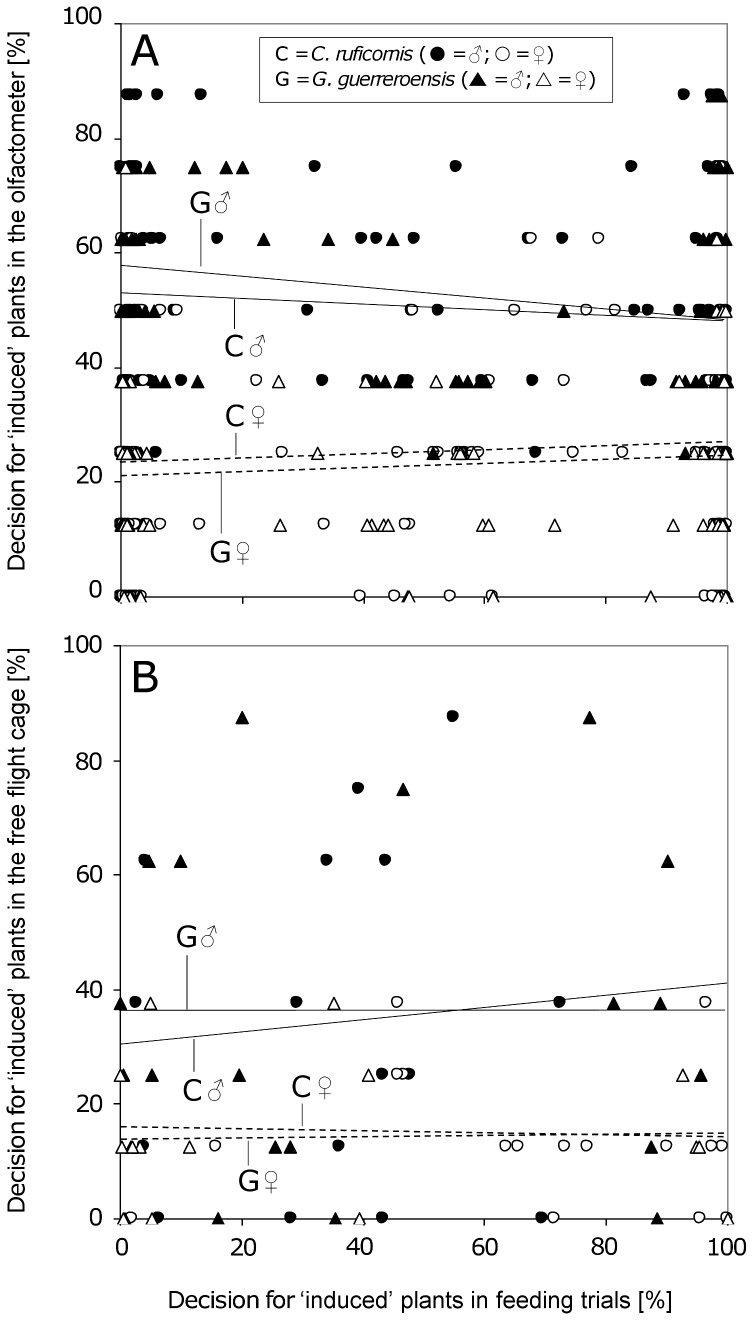
Correlation of decisions made in the olfactometer with feeding behavior. The percentage decision of individual male and female beetles of *C. ruficornis* (*n* = 16 males and *n* = 16 females) and *G. gynandrobrotica* (*n* = 19 males and *n* = 17 females) for induced plant material (JA treatments 1.0 and 0.001 mmol L^−1^; y-axis) in the olfactometer (A) and the free-flight cage (B) is displayed against the percentage decision for leaves of induced plants in feeding trials (x-axis). Percentage decision in feeding trials is calculated as the percentage of consumed leaf area of the induced leaf in relation to the overall leaf area consumption in the respective feeding trial ( = 100%). Pearson's correlations were used to evaluate associations between insects' decisions in the olfactometer and their feeding choice [A (as appearing in the figure from top to bottom): G♂: *r* = −0.151, *P* = 0.052; C♂: *r* = −0.091, *P* = 0.369; C♀: *r* = 0.064; *P* = 0.483; G♀: *r* = 0.084, *P* = 0.369; B (top to bottom): G♂: r = 0.086; *P* = 0.752; C♂: *r* = −0.123; *P* = 0.650; C♀: *r* = −0.008; *P* = 0.973; G♀: *r* = −0.049; *P* = 0.851].

**Figure 8:**
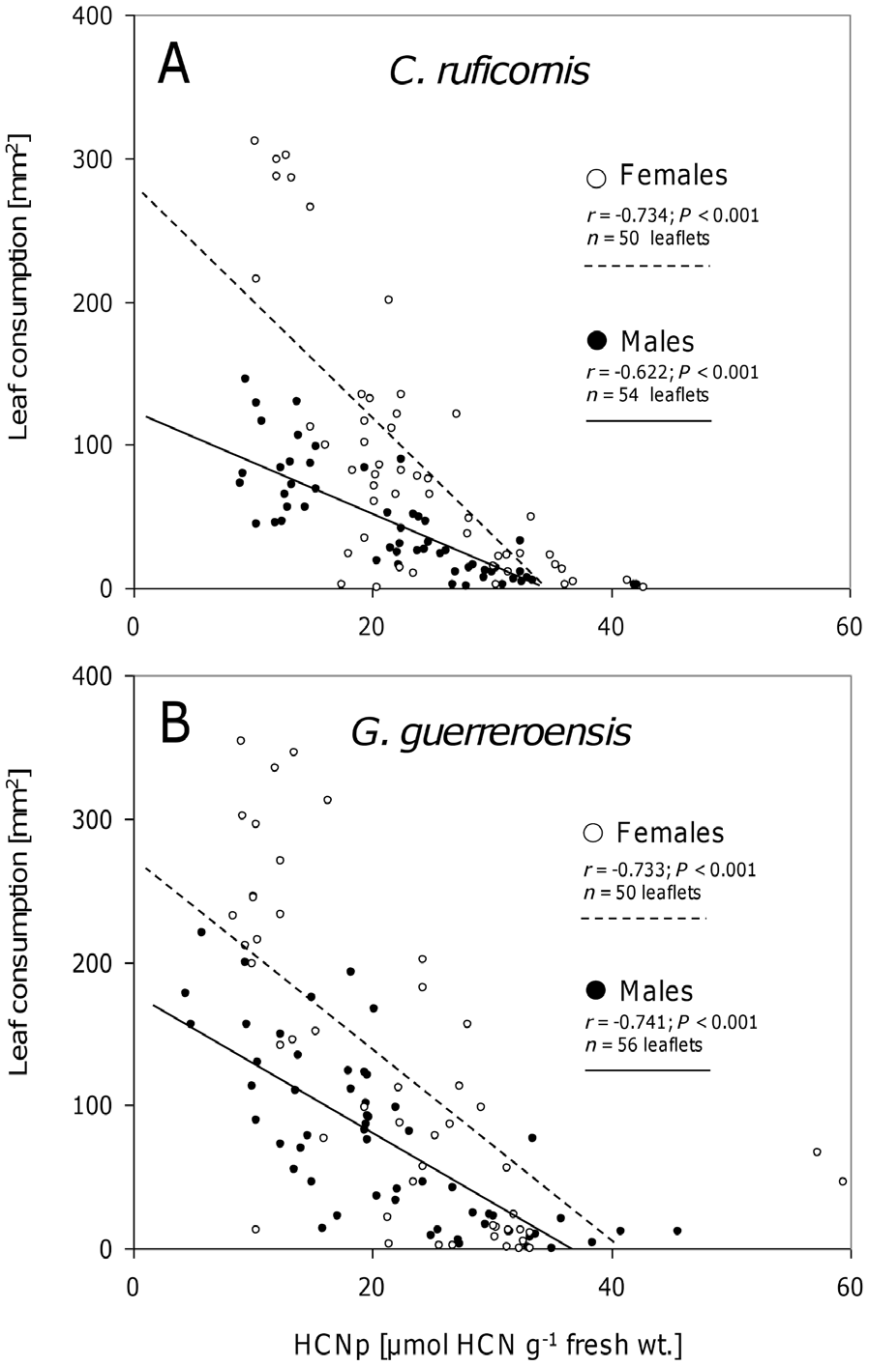
Leaf consumption of beetles in feeding trials with leaves from plants used in free-flight experiments. In binary-choice feeding experiments each two leaflets derived from plants previously used in free-flight experiments were offered to individual beetles of *Cerotoma ruficornis* (A) and *Gynandrobrotica guerreroensis* (B) and leaf area consumption was determined. Pearson's correlations were calculated to evaluate effects of HCNp on leaf consumption.

While jasmonic acid treatment had no effects on the feeding behavior of beetles (Fig. 7), the amount of leaf material consumed by both sexes was significantly negatively correlated to the cyanogenic potential (HCNp) (according to Pearson's correlation; Figs. 6 and 8). This negative correlation between HCNp and feeding choice was valid for leaves from mature shoots and intact young plants used in olfactometer experiments (Fig. 6) as well as for young plants used in the free-flight cage (Fig. 8). For both beetle species, females responded with higher sensitivity to HCNp than males, as indicated by the steeper inclination of the regression line (Figs. 6 and 8). Neither in olfactometer nor in free-flight experiments did we observe correlations between the VOC-mediated (long-distance) and HCNp-mediated (short-distance) choice behavior of male or female beetles of both species (Fig. 7A,B).

## Discussion

We investigated whether herbivorous beetles can use volatile organic compounds (VOCs) to localize host plants and whether long-distance responses towards these VOCs can serve as a reliable predictor of feeding choice. Supporting our initial predictions, we found that the release of JA-induced plant volatiles affected beetle behavior in a concentration- dependent manner. Female beetles were repelled by VOCs that had been induced both by high and low JA concentrations. Males, however, were repelled by the VOC spectra of plants treated with high JA concentrations, but were attracted to the VOCs released from plants treated with low JA- concentrations. Furthermore, we demonstrated that factors directly determining food quality are strong predictors of insect feeding decisions. The feeding behavior of both sexes was strongly determined by the cyanogenic potential of leaves. Again, females responded more sensitively than males.

For herbivorous insects, induced plant VOCs convey a broad range of information. In addition to indicating the mere presence of the plant, the specific chemical composition of VOCs can signal plant quality, as well as other vitally important factors such as, the presence of mating partners, competitors, or enemies [14], [15], [17]. Surprisingly, in comparison to the high number of studies on the effects of herbivore-induced volatiles on carnivorous arthropods, e.g., [31], [33], [38], herbivore behavior in response to induced plant volatiles has received little attention [6], [51]–[53]. Depending on the specific plant-herbivore system under study, findings range from attraction (e.g. *Chrysomela populi* on leaves of *Populus*×*euroamericana* [39]), to deterrence (e.g. *Heliothis virescens* on *Nicotiana tabacum* [52]).

In the present study, we used jasmonic acid (JA) at different concentrations to induce the release of VOCs in order to create significant variability in this trait without having VOCs in the system that might be directly derived from feeding herbivores. Cyanogenesis was determined as the most important direct defense that does not respond to JA treatment [28]. Interestingly, the sex of the beetles strongly affected their responses to VOCs: females always preferred non-induced controls whereas males preferred slightly induced plants—but not strongly induced ones—over controls (Fig. 3). The direct feeding decisions were, by contrast, significantly determined by the cyanogenic potential (HCNp) of leaves, with females responding more sensitively than males to different HCNp values (Fig. 6). Because leaf HCNp was not correlated with the JA-dependent release of VOCs, the long-distance orientation of the beetles—which was taken in response to VOCs—did not predict beetles' feeding decisions (Fig. 7). Although VOCs can be an important long-distance cue for herbivorous beetles, feeding decisions in our experiments were determined by a direct plant defense rather than VOCs.

The needs of an herbivorous insect to successfully locate and exploit a potential host plant are immense. Direct and indirect defenses as well as nutritional quality determine the potential success of a herbivore on a given host [54], [55]. Therefore, the interactions between plants and herbivores are often mediated by the combination of visual, olfactory and/or gustatory cues [9]. For example, chrysomelid herbivores have repeatedly been reported to respond to plant size [56], [57], or to silhouettes and color [54], [58]. Among olfactory cues, constitutively released VOCs as well as inducible VOCs can play a major role in attracting or deterring insect herbivores [51], [59]–[64]. Visual cues were excluded in our olfactometer experiments as the insects could not see the plant material offered. We also controlled for potential effects of plant age by using young, intact plants and shoots of large plants in the same experimental setup. Both plant developmental stages responded equally to all JA treatments except when induced with 1.0 mmol JA L^−1^ (Fig. 2). Overall beetle behavior in response to both plant developmental stages was very similar (Fig. 3). In the feeding trials, we exclusively used leaflets of a similar developmental stage to control for leaf age and size, a factor to which chrysomelids might respond sensitively [65]. Thus, the long-distance decisions of beetles we observed in the olfactometer were likely determined only by plant volatiles.

In both olfactometer and free-flight experiments, females always preferred non-induced plants, whereas males preferred slightly induced plants over controls and discriminated only against the most intensively induced plants. Interestingly, feeding damage invoked by *G. guerreroensis* induced VOC release similar to treatment with 0.01 and 0.001 mmol L^−1^ JA (Fig. 2). Plants damaged by this herbivore as well as plants treated with the respective JA concentrations attracted males, yet repelled females. We conclude that the induced release of plant VOCs represents a critical factor in the long-distance orientation of these beetles because released VOCs may indicate competition to females and thus, reduced resources for the developing offspring, whereas males might use such VOCs as indicators for the presence of putative mating partners.

However, why was the long-distance behavior not a reliable predictor for the feeding decisions of beetles, at least in the case of females? Although chrysomelids used in this study showed significant responses to the induced VOCs over long distances, the cyanogenic potential (HCNp) of leaflets was the crucial parameter that determined feeding choice, confirming earlier results [66]–[68]. Thus, the quantitative release of induced VOCs represented an efficient long-distance cue—both in olfactometer and free-flight experiments (Figs. 3 and 4)—whereas the leaf cyanide concentration determined the feeding behavior.

The amount of VOCs released in response to leaf damage by *Gynandrobrotica guerreroensis* paralleled the amount of VOCs released following plant treatments with JA solutions in concentrations of 0.001 and 0.01 mmol L^−1^ (Fig. 2). Female beetles of both species (*G. guerreroensis* and *Cerotoma ruficornis*) were repelled by VOCs at these concentrations, whereas male beetles were attracted to the induced plants (Fig. 3). However, the question arises whether or not the spectrum of VOCs released in response to JA treatments with higher concentrations of 0.1 and 1 mmol L^−1^ reflects the bouquet that can be released in response to natural insect feeding, or if the amount of the experimentally induced VOCs exceeds ecologically relevant limits. In fact, leaf feeding by Mexican bean beetles (Coccinellidae: *Epilachna varivestis*) resulted in amounts of volatiles which—despite substantial variation—frequently reached levels as seen following plant treatments with JA [6]. Thus, the amount of volatiles released in response to treatments with JA solutions at concentrations of 0.1 and 1.0 mmol L^−1^ can also occur in the plant-herbivore system.

At natural sites, both chrysomelid species have been observed to feed on a small range of wild and cultivated fabaceous plants (pers. observation by authors). However, lima beans are the preferred food plants and the insects can therefore be considered well adapted to feeding on this cyanogenic plant species [23], [69]. Generally, specialist or oligophagous herbivores with restricted host ranges are able to tolerate or detoxify defensive compounds of their respective host plants [70]. Nevertheless, whether or not specialists are quantitatively affected by cyanogenic features of their hosts is still uncertain [25]. Only few long-term experiments have studied the quantitative effects of cyanogenesis on parameters relevant to fitness, such as growth rates, developmental period, and success of reproduction itself [27], [71]. In addition to potential direct effects of cyanogenic precursors, indirect effects might also occur by slowing down the developmental rate of herbivores and thereby increasing their susceptibility to natural enemies—the ‘slow-growth-high-mortality hypothesis’ [54]. Thus, ecological costs arising directly or indirectly from feeding on highly cyanogenic lima bean individuals might explain why the cyanogenic potential ultimately determines feeding choice, even in well adapted herbivores [70].

Conversely, damaged plants indicate the potential presence of competitors and the induction of VOCs and extrafloral nectar attracts ants and other carnivores to lima bean in nature [72]. Therefore, females might avoid plants releasing high amount of VOCs, simply because these indicate a high predation pressure. Once on a plant, feeding behavior under our experimental conditions could not be affected by indirect defensive traits because predators had been excluded. HCNp turned out to be the most important determinant of plant quality for the adult beetles. For males, by contrast, the chance to find mating partners might outweigh the enhanced predation risk that is indicated by such VOCs, making the emitting plants attractive.

### Conclusions

Studies on the function of induced plant volatiles (VOCs) for host plant location of chrysomelid herbivores have yielded inconsistent results. Induced VOCs have been demonstrated to attract chrysomelid herbivores to their host plants [73], [74], while other studies have reported repellent effects [6], [38], [45]. Our present study suggests that the function of VOCs for locating and exploiting host plants is more complex than widely assumed. Responses of two chrysomelid herbivores (*Cerotoma ruficornis* and *Gynandrobrotica guerreroensis*) to herbivore-induced plant VOCs varied substantially depending on the amount of volatiles released and sex of the herbivores. Females were repelled by both low and high concentrations of VOCs, whereas males were repelled by high concentrations but attracted to plants releasing low amounts of VOCs. However, the information carried by VOCs must not necessarily reflect food plant quality, since feeding choice of insects was determined by the cyanogenic potential of leaves [65]. We conclude that VOCs might represent significant long-distance cues to locate host plants and assess other relevant factors such as the presence of potential mating partners, competitors, or enemies, whereas feeding behavior is determined by other plant traits that have a direct effect on the herbivore, such as, in the present case, direct plant defenses.

## Supporting Information

Table S1 **Treatment effects on the quantitative emission of volatile organic compounds (VOCs).** We statistically compared the VOC emission of tendrils with potted plants using Mann Whitney U-tests for each treatment. Plants were induced with different concentrations of jasmonic acid (JA), by feeding damage through herbivorous beetles (Feeding damage) or treated with water and served as control (Control).
(DOC)

Table S2 **Effect of induced plant volatiles on the choice behavior of** ***Cerotoma ruficornis*** **and** ***Gynandrobrotica guerreroensis*** **.** Decisions made by male and female beetles in olfactometer (mature lima bean shoots and young intact plants) and free flight experiments (young intact plants) were tested for significant differences by Wilcoxon signed rank tests. The plant material used in the choice experiments was induced for release of volatiles by different treatments: I (1.0) = 1.0 mmol L^−1^ jasmonic acid (JA); I (0.1) = 0.1 mmol L^−1^ JA; I (0.01) = 0.01 mmol L^−1^ JA; I (0.001) = 0.001 mmol L^−1^ JA; HI = herbivore-induced (*G. guerreroensis*) plant material. Plant material sprayed with water instead of JA (C) and empty olfactometer arms (0) served as controls.
(DOC)

Table S3 **Ontogenetic effects of plant volatiles on the choice behavior of** ***Cerotoma ruficornis*** **and** ***Gynandrobrotica guerreroensis*** **.** In olfactometer choice experiments with induced (sprayed with 1 mmol L^−1^ jasmonic acid) and untreated lima bean plants (mature shoots and young intact plants), decisions made by male and female beetles for the one or the other source were tested for significant differences with Wilcoxon signed rank tests. In these control experiments plant material of the same state of induction but various ontogenetic developmental stage was tested against each other.
(DOC)

Table S4 **Sex-specific choice behavior of** ***Cerotoma ruficornis*** **and** ***Gynandrobrotica guerreroensis*** **.** Choice behavior of beetles observed in olfactometer and free flight experiments using mature lima bean shoots and intact young plants as odor source was tested for sex-specific differences by Mann-Whitney U tests. Plant material used in the choice experiments was induced by various treatments: I (1.0) = spraying with 1.0 mmol L^−1^ jasmonic acid (JA); I (0.1) = 0.1 mmol L^−1^ JA; I (0.01) = 0.01 mmol L^−1^ JA; I (0.001) = 0.001 mmol L^−1^ JA; HI = herbivore-induced (*G. gynandrobrotica*) plant material. Untreated plant material (C) and empty olfactometer arms (0) served as controls.
(DOC)

Table S5 **Effects of ontogenetic plant develeopmental stage on sex-specific choice behavior of** ***Cerotoma ruficornis*** **and** ***Gynandrobrotica guerreroensis*** **.** Choice behavior of beetles observed in olfactometer experiments with induced (sprayed with 1 mmol L^−1^ jasmonic acid) and untreated mature lima bean shoots and intact young lima bean plants was tested for sex-specific differences with Mann-Whitney U tests. In these control experiments plant material of the same state of induction but various ontogenetic developmental stages was tested against each other.
(DOC)
