# Supplementary material for: Distance and Sex Determine Host Plant Choice by Herbivorous Beetles
Source: PLoS One. 2013 Feb 6;8(2):e55602. doi: 10.1371/journal.pone.0055602 (PMC3565971; doi:10.1371/journal.pone.0055602)
Supplement: Table S1 — Treatment effects on the quantitative emission of volatile organic compounds (VOCs). We statistically compared the VOC emission of tendrils with potted plants using Mann Whitney U-tests for each treatment. Plants were induced with different concentrations of jasmonic acid (JA), by feeding damage through herbivorous beetles (Feeding damage) or treated with water and served as control (Control). (DOC) [file pone.0055602.s001.doc]

| Responses of plant types upon induction | | | |  |  |
| --- | --- | --- | --- | --- | --- |
| Treatment |  | Total N | U | Z | P |
|  |  |  |  |  |  |
| JA induced (1.0 mmol L^-1^) |  | 18 | 66.000 | 2.252 | 0.024 |
| JA induced (0.1 mmol L^-1^) |  | 14 | 22.000 | -0.319 | 0.749 |
| JA induced (0.01 mmol L^-1^) |  | 14 | 25.000 | 0.064 | 0.949 |
| JA induced (0.001 mmol L^-1^) |  | 14 | 28.000 | 0.447 | 0.655 |
| Feeding damage |  | 14 | 27.000 | 0.319 | 0.749 |
| Control |  | 25 | 87.000 | 0.666 | 0.506 |
|  |  |  |  |  |  |
