# Supplementary material for: Distance and Sex Determine Host Plant Choice by Herbivorous Beetles
Source: PLoS One. 2013 Feb 6;8(2):e55602. doi: 10.1371/journal.pone.0055602 (PMC3565971; doi:10.1371/journal.pone.0055602)
Supplement: Table S2 — Effect of induced plant volatiles on the choice behavior of Cerotoma ruficornis and Gynandrobrotica guerreroensis . Decisions made by male and female beetles in olfactometer (mature lima bean shoots and young intact plants) and free flight experiments (young intact plants) were tested for significant differences by Wilcoxon signed rank tests. The plant material used in the choice experiments was induced for release of volatiles by different treatments: I (1.0) = 1.0 mmol L−1 jasmonic acid (JA); I (0.1) = 0.1 mmol L−1 JA; I (0.01) = 0.01 mmol L−1 JA; I (0.001) = 0.001 mmol L−1 JA; HI = herbivore-induced (G. guerreroensis) plant material. Plant material sprayed with water instead of JA (C) and empty olfactometer arms (0) served as controls. (DOC) [file pone.0055602.s002.doc]

| Olfactometer choice experiments | | | |  |  |
| --- | --- | --- | --- | --- | --- |
| Experimental setup |  |  | |  |  |
| **A** (Mature shoots + *C. ruficornis*) |  | N | W | Z | P |
|  |  |  |  |  |  |
| I (1.0):C (male) |  | 11 | 41.000 | 2.239 | 0.025 |
| I (1.0):C (female) |  | 17 | 153.000 | 3.676 | < 0.001 |
| I (0.1):C (male) |  | 10 | 25.000 | 0.303 | 0.762 |
| I (0.1):C (female) |  | 16 | 136.000 | 3.595 | < 0.001 |
| I (0.01):C (male) |  | 11 | 7.500 | -0.647 | 0.518 |
| I (0.01):C (female) |  | 19 | 150.000 | 3.532 | < 0.001 |
| I (0.001):C (male) |  | 14 | 0.000 | -3.097 | 0.002 |
| I (0.001):C (female) |  | 12 | 66.000 | 2.971 | 0.003 |
| HI:C (male) |  | 11 | 0.000 | -2.565 | 0.010 |
| HI:C (female) |  | 15 | 120.000 | 3.427 | 0.001 |
| 0:C (male) |  | 6 | 21.000 | 2.232 | 0.026 |
| 0:C (female) |  | 7 | 21.000 | 2.226 | 0.026 |
| 0:0 (male) |  | 6 | 0.000 | -1.000 | 0.317 |
| 0:0 (female) |  | 8 | 3.000 | 1.414 | 0.157 |
|  |  |  |  |  |  |
| **B** (Young plants + *C. ruficornis*) |  |  |  |  |  |
|  |  |  |  |  |  |
| I (1.0):C (male) |  | 16 | 69.000 | 2.390 | 0.017 |
| I (1.0):C (female) |  | 15 | 91.000 | 3.213 | < 0.001 |
| I (0.1):C (male) |  | 12 | 40.500 | 2.309 | 0.051 |
| I (0.1):C (female) |  | 15 | 120.000 | 3.436 | 0.001 |
| I (0.01):C (male) |  | 11 | 2.500 | -2.209 | 0.027 |
| I (0.01):C (female) |  | 16 | 101.500 | 3.128 | 0.002 |
| I (0.001):C (male) |  | 14 | 0.000 | -2.848 | 0.004 |
| I (0.001):C (female) |  | 13 | 66.000 | 2.971 | 0.003 |
| HI:C (male) |  | 12 | 3.000 | -2.565 | 0.010 |
| HI:C (female) |  | 15 | 120.000 | 3.436 | < 0.001 |
| 0:C (male) |  | 6 | 21.000 | 2.232 | 0.026 |
| 0:C (female) |  | 6 | 21.000 | 2.264 | 0.024 |
| 0:0 (male) |  | 6 | 3.000 | 1.414 | 0.157 |
| 0:0 (female) |  | 6 | 0.000 | -1.000 | 0.317 |
|  |  |  |  |  |  |
| **C** (Mature shoots + *G. guerreroensis*) |  |  |  |  |  |
|  |  |  |  |  |  |
| I (1.0):C (male) |  | 14 | 47.000 | 2.040 | 0.041 |
| I (1.0):C (female) |  | 14 | 105.000 | 3.355 | 0.001 |
| I (0.1):C (male) |  | 8 | 10.000 | 0.707 | 0.480 |
| I (0.1):C (female) |  | 10 | 55.000 | 2.829 | 0.005 |
| I (0.01):C (male) |  | 12 | 22.500 | 0.000 | 1.000 |
| I (0.01):C (female) |  | 18 | 150.500 | 3.555 | < 0.001 |
| I (0.001):C (male) |  | 12 | 0.000 | -3.002 | 0.003 |
| I (0.001):C (female) |  | 14 | 78.000 | 3.088 | 0.002 |
| HI:C (male) |  | 9 | 0.000 | -2.558 | 0.011 |
| HI:C (female) |  | 16 | 136.000 | 3.559 | < 0.001 |
| 0:C (male) |  | 7 | 21.000 | 2.232 | 0.026 |
| 0:C (female) |  | 6 | 21.000 | 2.226 | 0.026 |
| 0:0 (male) |  | 8 | 2.000 | -0.577 | 0.564 |
| 0:0 (female) |  | 6 | 2.000 | -0.577 | 0.565 |
|  |  |  |  |  |  |
| **D** (Young plants + *G. guerreroensis*) |  |  |  |  |  |
|  |  |  |  |  |  |
| I (1.0):C (male) |  | 14 | 90.000 | 2.428 | 0.014 |
| I (1.0):C (female) |  | 14 | 90.500 | 3.015 | 0.003 |
| I (0.1):C (male) |  | 10 | 24.500 | 1.897 | 0.058 |
| I (0.1):C (female) |  | 16 | 136.000 | 3.559 | < 0.001 |
| I (0.01):C (male) |  | 12 | 0.000 | -2.428 | 0.055 |
| I (0.01):C (female) |  | 15 | 120.000 | 3.457 | 0.001 |
| I (0.001):C (male) |  | 13 | 0.000 | -3.140 | 0.002 |
| I (0.001):C (female) |  | 14 | 78.000 | 3.088 | 0.002 |
| HI:C (male) |  | 11 | 6.000 | -2.228 | 0.026 |
| HI:C (female) |  | 16 | 136.000 | 3.539 | < 0.001 |
| 0:C (male) |  | 5 | 15.000 | 2.032 | 0.042 |
| 0:C (female) |  | 6 | 21.000 | 2.214 | 0.027 |
| 0:0 (male) |  | 10 | 3.500 | -0.552 | 0.581 |
| 0:0 (female) |  | 8 | 10.000 | 0.707 | 0.480 |
|  |  |  |  |  |  |
| Free flight choice experiments | | | |  |  |
| Experimental setup |  |  |  |  |  |
| **A** (Young plants + *C. ruficornis*) |  | N | W | Z | P |
|  |  |  |  |  |  |
| I (1.0):C (male) |  | 7 | 28.000 | 2.366 | 0.018 |
| I (1.0):C (female) |  | 9 | 36.000 | 2.535 | 0.001 |
| I (0.001):C (male) |  | 7 | 0.000 | -2.366 | 0.008 |
| I (0.001):C (female) |  | 7 | 28.000 | 2.375 | 0.008 |
| HI:C (male) |  | 8 | 0.000 | -2.527 | 0.002 |
| HI:C (female) |  | 8 | 28.000 | 2.366 | 0.008 |
| C:C (male) |  | 11 | 16.000 | -0.772 | 0.440 |
| C:C (female) |  | 9 | 9.500 | -0.210 | 0.833 |
|  |  |  |  |  |  |
| **B** (Young plants + *G. guerreroensis*) |  |  |  |  |  |
|  |  |  |  |  |  |
| I (1.0):C (male) |  | 10 | 45.000 | 2.673 | 0.018 |
| I (1.0):C (female) |  | 8 | 27.000 | 2.201 | 0.028 |
| I (0.001):C (male) |  | 9 | 0.000 | -2.673 | 0.008 |
| I (0.001):C (female) |  | 9 | 45.000 | 2.670 | 0.008 |
| HI:C (male) |  | 9 | 0.000 | -2.527 | 0.006 |
| HI:C (female) |  | 9 | 45.000 | 2.670 | 0.008 |
| C:C (male) |  | 9 | 6.000 | -0.946 | 0.344 |
| C:C (female) |  | 8 | 22.500 | 0.634 | 0.526 |
|  |  |  |  |  |  |
