# Supplementary material for: Distance and Sex Determine Host Plant Choice by Herbivorous Beetles
Source: PLoS One. 2013 Feb 6;8(2):e55602. doi: 10.1371/journal.pone.0055602 (PMC3565971; doi:10.1371/journal.pone.0055602)
Supplement: Table S3 — Ontogenetic effects of plant volatiles on the choice behavior of Cerotoma ruficornis and Gynandrobrotica guerreroensis . In olfactometer choice experiments with induced (sprayed with 1 mmol L−1 jasmonic acid) and untreated lima bean plants (mature shoots and young intact plants), decisions made by male and female beetles for the one or the other source were tested for significant differences with Wilcoxon signed rank tests. In these control experiments plant material of the same state of induction but various ontogenetic developmental stage was tested against each other. (DOC) [file pone.0055602.s003.doc]

| Olfactometer choice experiments | | | |  |  |
| --- | --- | --- | --- | --- | --- |
| Experimental setup |  |  | |  |  |
| **A** (Shoots/young plants + *C. ruficornis*) |  | N | W | Z | P |
|  |  |  |  |  |  |
| Shoots I (1.0): Shoots I (1.0) (male) |  | 7 | 11.500 | 1.089 | 0.276 |
| Shoots I (1.0): Shoots I (1.0) (female) |  | 6 | 3.500 | 0.272 | 0.785 |
| Young plants I (1.0): Young plants I (1.0) (male) |  | 5 | 1.500 | -1.300 | 0.194 |
| Young plants I (1.0): Young plants I (1.0) (female) |  | 4 | 0.000 | -1.000 | 0.317 |
| Shoots C: Shoots C (male) |  | 6 | 1.500 | 0.000 | 1.000 |
| Shoots C: Shoots C (female) |  | 8 | 7.500 | 1.000 | 0.317 |
| Young plants C: Young plants C (male) |  | 10 | 20.000 | 1.035 | 0.301 |
| Young plants C: Young plants C (female) |  | 8 | 3.000 | -0.736 | 0.461 |
| Shoots I (1.0): Young plants I (1.0) (male) |  | 12 | 18.000 | 0.000 | 1.000 |
| Shoots I (1.0): Young plants I (1.0) (female) |  | 12 | 27.000 | 1.414 | 0.151 |
| Shoots C: Young plants C (male) |  | 9 | 14.000 | 0.755 | 0.450 |
| Shoots C: Young plants C (female) |  | 9 | 10.000 | -0.690 | 0.490 |
|  |  |  |  |  |  |
| **B** (Shoots/young plants + *G. guerreroensis*) |  |  |  |  |  |
|  |  |  |  |  |  |
| Shoots I (1.0): Shoots I (1.0) (male) |  | 7 | 1.000 | -1.069 | 0.285 |
| Shoots I (1.0): Shoots I (1.0) (female) |  | 6 | 1.500 | 0.000 | 1.000 |
| Young plants I (1.0): Young plants I (1.0) (male) |  | 6 | 1.000 | 1.000 | 0.317 |
| Young plants I (1.0): Young plants I (1.0) (female) |  | 4 | 3.500 | 0.272 | 0.785 |
| Shoots C: Shoots C (male) |  | 5 | 1.500 | 0.000 | 1.000 |
| Shoots C: Shoots C (female) |  | 5 | 1.500 | 0.000 | 1.000 |
| Young plants C: Young plants C (male) |  | 10 | 12.000 | -0.347 | 0.729 |
| Young plants C: Young plants C (female) |  | 8 | 6.000 | 0.378 | 0.705 |
| Shoots I (1.0): Young plants I (1.0) (male) |  | 13 | 35.500 | 0.223 | 0.823 |
| Shoots I (1.0): Young plants I (1.0) (female) |  | 9 | 1.500 | -1.625 | 0.104 |
| Shoots C: Young plants C (male) |  | 12 | 10.500 | -0.604 | 0.546 |
| Shoots C: Young plants C (female) |  | 6 | 17.500 | 1.510 | 0.131 |
|  |  |  |  |  |  |
