# Supplementary material for: Distance and Sex Determine Host Plant Choice by Herbivorous Beetles
Source: PLoS One. 2013 Feb 6;8(2):e55602. doi: 10.1371/journal.pone.0055602 (PMC3565971; doi:10.1371/journal.pone.0055602)
Supplement: Table S4 — Sex-specific choice behavior of Cerotoma ruficornis and Gynandrobrotica guerreroensis . Choice behavior of beetles observed in olfactometer and free flight experiments using mature lima bean shoots and intact young plants as odor source was tested for sex-specific differences by Mann-Whitney U tests. Plant material used in the choice experiments was induced by various treatments: I (1.0) = spraying with 1.0 mmol L−1 jasmonic acid (JA); I (0.1) = 0.1 mmol L−1 JA; I (0.01) = 0.01 mmol L−1 JA; I (0.001) = 0.001 mmol L−1 JA; HI = herbivore-induced (G. gynandrobrotica) plant material. Untreated plant material (C) and empty olfactometer arms (0) served as controls. (DOC) [file pone.0055602.s004.doc]

| Olfactometer choice experiments |  |  | |  |  |
| --- | --- | --- | --- | --- | --- |
| Experimental setup |  |  | |  |  |
| **A** (Mature shoots + *C. ruficornis*) |  | Total N | U | Z | P |
|  |  |  |  |  |  |
| I (1.0):C |  | 28 | 169.500 | -3.720 | < 0.001 |
| I (0.1):C |  | 26 | 6.000 | -4.059 | < 0.001 |
| I (0.01):C |  | 30 | 25.500 | -3.478 | < 0.001 |
| I (0.001):C |  | 26 | 1.000 | -4.316 | < 0.001 |
| HI:C |  | 26 | 0.000 | -4.320 | < 0.001 |
| 0:C |  | 13 | 16.500 | -0.699 | 0.485 |
| 0:0 |  | 10 | 10.500 | -0.454 | 0.655 |
|  |  |  |  |  |  |
| **B** (Young plants + *C. ruficornis*) |  |  |  |  |  |
|  |  |  |  |  |  |
| I (1.0):C |  | 31 | 49.500 | -2.835 | 0.005 |
| I (0.1):C |  | 27 | 20.000 | -3.563 | < 0.001 |
| I (0.01):C |  | 23 | 19.000 | -2.932 | 0.003 |
| I (0.001):C |  | 27 | 4.000 | -4.278 | < 0.001 |
| HI:C |  | 27 | 2.500 | -4.327 | < 0.001 |
| 0:C |  | 12 | 16.500 | -0.251 | 0.801 |
| 0:0 |  | 14 | 15.000 | -1.620 | 0.105 |
|  |  |  |  |  |  |
| **C** (Mature shoots + *G. guerreroensis*) |  |  |  |  |  |
|  |  |  |  |  |  |
| I (1.0):C |  | 28 | 21.000 | -3.632 | < 0.001 |
| I (0.1):C |  | 18 | 5.500 | -3.122 | 0.002 |
| I (0.01):C |  | 30 | 23.500 | -3.643 | < 0.001 |
| I (0.001):C |  | 29 | 3.000 | -4.507 | < 0.001 |
| HI:C |  | 24 | 0.000 | -4.100 | < 0.001 |
| 0:C |  | 13 | 12.000 | -1.344 | 0.179 |
| 0:0 |  | 14 | 25.000 | 0.145 | 0.885 |
|  |  |  |  |  |  |
| **D** (Young plants + *G. guerreroensis*) |  |  |  |  |  |
|  |  |  |  |  |  |
| I (1.0):C |  | 28 | 46.000 | -2.480 | 0.013 |
| I (0.1):C |  | 26 | 14.000 | -3.593 | < 0.001 |
| I (0.01):C |  | 27 | 0.000 | -4.483 | < 0.001 |
| I (0.001):C |  | 27 | 1.000 | -4.431 | < 0.001 |
| HI:C |  | 27 | 5.000 | -4.143 | < 0.001 |
| 0:C |  | 11 | 16.500 | 0.295 | 0.768 |
| 0:0 |  | 14 | 23.000 | -0.153 | 0.879 |
|  |  |  |  |  |  |
| Free flight choice experiments |  |  |  |  |  |
| Experimental setup |  |  |  |  |  |
| **A** (Young plants + *C. ruficornis*) |  | Total N | U | Z | P |
|  |  |  |  |  |  |
| I (1.0):C |  | 16 | 38.500 | 0.797 | 0.426 |
| I (0.001):C |  | 14 | 2.500 | -2.862 | 0.004 |
| HI:C |  | 16 | 1.500 | -3.256 | < 0.001 |
| C:C |  | 20 | 50.000 | 0.039 | 0.969 |
|  |  |  |  |  |  |
| **B** (Young plants + *G. guerreroensis*) |  |  |  |  |  |
|  |  |  |  |  |  |
| I (1.0):C |  | 18 | 37.000 | -0.279 | 0.780 |
| I (0.001):C |  | 18 | 4.500 | -3.219 | < 0.001 |
| HI:C |  | 20 | 10.000 | -3.065 | 0.002 |
| C:C |  | 17 | 41.000 | 0.497 | 0.619 |
|  |  |  |  |  |  |
