# Supplementary material for: Distance and Sex Determine Host Plant Choice by Herbivorous Beetles
Source: PLoS One. 2013 Feb 6;8(2):e55602. doi: 10.1371/journal.pone.0055602 (PMC3565971; doi:10.1371/journal.pone.0055602)
Supplement: Table S5 — Effects of ontogenetic plant develeopmental stage on sex-specific choice behavior of Cerotoma ruficornis and Gynandrobrotica guerreroensis . Choice behavior of beetles observed in olfactometer experiments with induced (sprayed with 1 mmol L−1 jasmonic acid) and untreated mature lima bean shoots and intact young lima bean plants was tested for sex-specific differences with Mann-Whitney U tests. In these control experiments plant material of the same state of induction but various ontogenetic developmental stages was tested against each other. (DOC) [file pone.0055602.s005.doc]

| Olfactometer choice experiments |  |  | |  |  |
| --- | --- | --- | --- | --- | --- |
| Experimental setup |  |  | |  |  |
| **A** (Shoots/young plants + *C. ruficornis*) |  | Total N | U | Z | P |
|  |  |  |  |  |  |
| Shoots I (1.0): Shoots I (1.0) |  | 9 | 15.000 | 1.291 | 0.197 |
| Young plants I (1.0): Young plants I (1.0) |  | 9 | 8.500 | -0.391 | 0.696 |
| Shoots C: Shoots C |  | 14 | 26.000 | 0.303 | 0.762 |
| Young plants C: Young plants C |  | 18 | 49.000 | 0.844 | 0.399 |
| Shoots I (1.0): Young plants I (1.0) |  | 24 | 58.000 | -0.866 | 0.387 |
| Shoots C: Young plants C |  | 18 | 52.500 | 1.098 | 0.272 |
|  |  |  |  |  |  |
|  |  |  |  |  |  |
| **B** (Shoots/young plants + *G. guerreroensis*) |  |  |  |  |  |
|  |  |  |  |  |  |
| Shoots I (1.0): Shoots I (1.0) |  | 13 | 18.500 | -0.411 | 0.681 |
| Young plants I (1.0): Young plants I (1.0) |  | 10 | 16.500 | 1.369 | 0.171 |
| Shoots C: Shoots C |  | 10 | 12.000 | -0.118 | 0.906 |
| Young plants C: Young plants C |  | 18 | 28.500 | -1.083 | 0.279 |
| Shoots I (1.0): Young plants I (1.0) |  | 22 | 88.500 | 2.091 | 0.037 |
| Shoots C: Young plants C |  | 18 | 16.000 | -1.972 | 0.049 |
|  |  |  |  |  |  |
